# A pre-operative prognostic score for the selection of patients for salvage surgery after recurrent head and neck squamous cell carcinomas

**DOI:** 10.1038/s41598-020-79759-0

**Published:** 2021-01-12

**Authors:** Valentina Lupato, Jerry Polesel, Fabio Biagio La Torre, Giuseppe Fanetti, Elisabetta Fratta, Carlo Gobitti, Gustavo Baldassarre, Emanuela Vaccher, Giovanni Franchin, Vittorio Giacomarra

**Affiliations:** 1 Division of Otolaryngology, General Hospital “Santa Maria Degli Angeli”, Pordenone, Italy; 2 Unit of Cancer Epidemiology, Centro di Riferimento Oncologico di Aviano (CRO) IRCCS, Aviano, Italy; 3 Division of Radiotherapy, Centro di Riferimento Oncologico di Aviano (CRO) IRCCS, Aviano, Italy; 4 Division of Immunopathology and Cancer Biomarkers, Centro di Riferimento Oncologico di Aviano (CRO) IRCCS, Aviano, Italy; 5 Division of Molecular Oncology, Centro di Riferimento Oncologico di Aviano (CRO) IRCCS, Aviano, Italy; 6 Division of Medical Oncology, Centro di Riferimento Oncologico di Aviano (CRO) IRCCS, Aviano, Italy

**Keywords:** Cancer, Head and neck cancer

## Abstract

Salvage surgery in recurrent head and neck squamous cell carcinoma has a poor outcome, both in terms of survival and quality of life. Therefore, the identification of pre-operative prognostic factors to improve the selection of patients who could benefit the most from salvage surgery is clinically relevant. The present study is a single-center retrospective analysis of 164 patients treated with salvage surgery after recurrence of head and neck cancer. Progression free survival and overall survival were calculated through Kaplan–Meier method. Hazard risk (HR) and corresponding confidence intervals (CI) were estimated through Cox proportional hazard model, adjusting for potential confounders. Significant predictors were combined into a prognostic score, attributing one point to each factor. Progression-free survival and overall survival were respectively 50.3% and 56.5% at 2 years, and 36.6% and 44.2% at 5 years. Four pre-operative factors were independently associated with poor prognosis: age > 70 years (HR = 2.18; 95% CI 1.27–3.73), initial stage IV (HR = 2.37; 95% CI 1.18–4.76), disease free interval < 12 months (HR = 1.72; 95% CI 1.01–2.94), and loco-regional recurrence (HR = 2.22; 95% CI 1.22–4.04). No post operative factor was associated with oncologic outcomes. Patients with 3–4 unfavorable factors showed a 5-year overall survival of 0.0% compared to 65.7% in those with 0–1 unfavorable factors (HR = 5.61; 95% CI 2.89–10.92). Despite the low number of patients, 3–4 unfavorable factors were associated to worse prognosis in all sub-sites. In conclusion, age > 70 years, initial stage IV, disease-free interval < 12 months, and loco-regional recurrence are strong independent pre-operative predictors of poor outcome in patients undergoing salvage surgery. Patients with two or more of these factors should be informed about the low success rate after salvage surgery and alternative treatments should be considered.

## Introduction

Despite the elective treatment, 25–60% of patients with an advanced stage head and neck squamous cell carcinoma (HNSCC) develops a recurrence^1,2^. In these scenarios, salvage surgery is generally considered the best curative option for resectable disease^3^. However, surgical treatment of locally recurrent HNSCC is challenging most of the times. In fact, wide excisions are often required to achieve negative margins, increasing the risk of functional and esthetic consequences.

Together with the patient’s general condition, the real tumor extension in the previously treated field, unusual pattern of local and regional spread, fibrosis, ischemia, and high rate of post operative complications should be evaluated before salvage surgery^4,5^. Indeed, the post-operative complications rate is generally high, especially in patients previously treated with radiotherapy associated or not with chemotherapy^6^. To this regard, it is important to highlight that, in the last two decades, radiotherapy with or without concurrent chemotherapy, has been established as primary treatment for a variety of sub-sites and stages of HNSCC, except those of the oral cavity^7^.

Considering that 25–50% of operated patients develops a second recurrence after salvage treatment and that the overall survival is quite low^8^, quality of residual life assumes an enormous importance^9^. A multidisciplinary team of skilled professionals should select patients for salvage surgery on agreed evidence-based guidelines, and the decision should involve a well-informed patient. In this context, preoperative prognostic factors would be helpful to identify patients at risk of poor outcome after salvage surgery.

So far, several pre-operative factors have been reported as prognostic indicators in recurrent HNSCCs, including initial stage, stage at recurrence, site, performance status, age, primary treatment (surgery versus chemo/radiotherapy), HPV status, lymph nodes involvement, disease-free interval (DFI), and loco-regional recurrence. Furthermore, status of resection margins, extracapsular invasion and post-operative complications have been identified as potential post-operative prognostic factors^3^.

Despite these findings, studies comparing pre- and post-operative factors are lacking. Furthermore, the paucity of cases in single studies weakens statistical analysis. Based on these considerations, the aim of this study was to evaluate pre- and post-operative factors in order to develop a prognostic score that might improve the selection of patients who could benefit the most from salvage surgery.

## Methods

We retrospectively retrieved data from 166 eligible patients who fulfilled the following inclusion criteria: (a) patients with primary diagnosis of HNSCC; (b) patients’ initial treatment with curative intent by radiotherapy alone, radiotherapy with concurrent chemotherapy, surgery alone, or surgery followed by adjuvant chemo-radiotherapy; and (c) patients who underwent salvage surgery for local, regional or loco-regional recurrence between January 2006 and December 2018. Two patients were lost to follow-up, thus leaving 164 patients for the present analysis. Since 2005, patients who underwent radiotherapy where treated with intensity modulated radiation therapy (IMRT). The study protocol was approved by the Regional Board of Ethics (Protocol number: CRO-2019-65), confirming that all research was performed in accordance with relevant guidelines and regulations for retrospective studies. Alive enrolled patients provided a written informed consent for the use of clinical data for research purpose. According to the authorization 9/2016 (art. 4) by the Italian Data Protection Authority, informed consent for deceased patients is no longer a requirement in retrospective studies, once the study protocol has been approved by the Board of Ethics.

An experienced multidisciplinary team of physicians including head and neck surgeons, dedicated radiation oncologists, and medical oncologists evaluated all patients. Diagnostic and therapeutic decisions were made according to internal protocols based on international guide-lines. Loco-regional assessment included fiberoptic nasolaryngoscopy, biopsy under local or general anesthesia, and head and neck contrast-enhanced computed tomography ± magnetic resonance imaging. Distant metastases were ruled out with computed tomography scan of the chest and, in advanced stages, with F-18-fluorodeoxyglucose positron emission tomography/computer tomography. Patients were staged or restaged according the UICC TNM classification system, seventh edition^10^.

Major surgical complications were retrieved from medical records. Angiolymphatic invasion and perineural invasion were assessed in the patients who underwent salvage surgery on tumor site recurrence. Margins of resection were evaluated on the surgical specimen and were defined as “close” when measuring less than 5 mm, and positive when measuring less than 1 mm. Nodal extracapsular spread was also collected and defined as positive margin.

Baseline characteristics are presented as proportions and differences across groups were evaluated through Fisher’s exact test. For each patient, person-time at risk was computed from the date of salvage surgery to the event date or the date of last follow-up, whichever came first. Event was defined as cancer recurrence or death for PFS or death for OS. The Kaplan–Meier method was used to generate crude survival curves and the log-rank test was used to assess the heterogeneity in time to event in strata of selected covariates^11^, censoring follow-up at 5 years. Hazard ratios (HR) and the corresponding 95% CI were calculated using Cox proportional hazards models^11^, adjusting for covariates significantly associated with OS in the univariate analysis (i.e., age, primary cancer site, initial TNM stage, DFI, recurrence site). Parameters’ significance was tested through Wald χ^2^ test^11^.

The four factors independently associated with OS—i.e., the factors significantly associated with OS in the multivariable model— (namely, age ≥ 70 years, stage IV, DFI < 12 months, and loco-regional recurrence) were integrated into a predicting score. Considering that these predictors showed a similar association, 1 point was assigned to each factor, so that the score summed up to a maximum of 4 points. Score prognostic value was compared with previous scores^1,12,13^, using Harrell’s C-index^14^. Statistical significance was claimed for P < 0.05 (two-sided). Analysis were performed with SAS 9.4.

## Results

### Study patients

Patients recurred after a median DFI of 7 months (interquartile range: 4–32 months). The majority of patients (n = 90, 54.9%) reported local recurrence, whereas isolated regional recurrence and loco-regional recurrence were diagnosed in 48 and 26 patients, respectively (Table 1). Local recurrence occurred more frequently in patients with laryngeal cancer than at other sites, with initial stage I-II than higher stages, and in those who recurred after a longer DFI. Conversely, regional or loco-regional recurrence was more frequent in patients with oropharyngeal cancers and in those with initial lymph nodes involvement (Table 1). Seventy-three (44.5%) patients underwent surgery on the tumor site with neck dissection, 38 patients (23.1%) underwent salvage surgery on the tumor site exclusively, and 53 (32.3%) patients underwent neck dissection alone.

**Table 1:** Distribution of 164 patients undergoing salvage surgery after recurrent head and neck cancer, according to sociodemographic and pre-operative clinical characteristics.

	Overall		Recurrence					
			Local		Regional		Loco-regional	
	n	(%)	n	(%)	n	(%)	n	(%)
**Gender**								
Male	122	(74.4)	70	(77.8)	30	(62.5)	22	(84.6)
Female	42	(25.6)	20	(22.2)	18	(37.5)	4	(15.4)
Fisher exact test			P = 0.068					
**Age at salvage surgery (year)**^**a**^								
< 60	61	(37.7)	32	(36.4)	20	(41.7)	9	(34.6)
60–69	57	(35.2	36	(40.9)	12	(25.0)	9	(34.6)
≥ 70	44	(27.2)	20	(22.7)	16	(33.3)	8	(30.8)
Fisher exact test			P = 0.393					
**Primary cancer site**								
Oral cavity	40	(24.4)	21	(23.3)	16	(33.3)	3	(11.5)
Oropharynx	54	(32.9)	20	(22.2)	22	(45.8)	12	(46.2)
Hypopharynx	25	(15.2)	13	(14.4)	5	(10.4)	7	(26.9)
Larynx	45	(27.4)	36	(40.0)	5	(10.4)	4	(15.4)
Fisher exact test			P < 0.001					
**Initial T stage**								
T1-T2	76	(46.3)	45	(50.0)	24	(50.0)	7	(26.9)
T3-T4	56	(34.1)	28	(31.1)	16	(33.3)	12	(46.2)
Unknown	32	(19.5)	17	(18.9)	8	(16.7)	7	(26.9)
Fisher exact test			P = 0.283					
**Initial N stage**								
N0	60	(36.6)	47	(52.2)	8	(16.7)	5	(19.2)
N1-N3	67	(40.9)	22	(24.4)	31	(64.6)	14	(53.9)
Unknown	37	(22.6)	21	(23.3)	9	(18.8)	7	(26.9)
Fisher exact test			P < 0.001					
**Initial TNM stage**								
I-II	45	(27.4)	37	(41.1)	6	(12.5)	2	(7.7)
III	34	(20.7)	19	(21.1)	9	(18.8)	6	(23.1)
IV	53	(32.3)	17	(18.9)	25	(52.1)	11	(42.3)
Unknown	32	(19.5)	17	(18.9)	8	(16.7)	7	(26.9)
Fisher exact test			P < 0.001					
**Initial treatment**								
Surgery ± RT/CRT	39	(23.8)	21	(23.3)	14	(29.2)	4	(15.4)
RT ± CT	125	(76.2)	69	(76.7)	34	(70.8)	22	(84.6)
Fisher exact test			P = 0.451					
**Disease-free interval (months)**^**a**^								
< 6	57	(38.8)	20	(24.4)	27	(65.9)	10	(41.7)
6–12	34	(23.1)	20	(24.4)	7	(17.1)	7	(29.2)
≥ 12	56	(38.1)	42	(51.2)	7	(17.1)	7	(29.2)
Fisher exact test			P < 0.001					

*RT* radiotherapy, *CT* chemotherapy, *CRT* chemo-radiotherapy.

^a^The sum does not add up to total because of missing values.

Considering 111 patients treated with salvage surgery on primary site, we performed 22 (19.8%) total laryngectomies, 21 (18.9%) total pharyngo-laryngectomies, 21 (18.9%) cordectomies/partial laryngectomies, 15 (13.1%) oropharyngectomies, 10 (9.0%) trans-oral resections, 8 (7.2%) mandibulectomies, 7 (6.3%) total glosso-laryngectomies, 7 (6.3%) total/partial glossectomies. Reconstruction using distant pedicled and microvascular free flaps was performed in 54 (48.6%) patients.

### Pre-operative prognostic factors

After a median follow-up of 26 months (interquartile rage: 11–73 months) from salvage surgery, 98 deaths were observed, leading to an OS of 56.5% at 2 years and of 44.2% at 5 years. PFS was 50.3% and 36.6%, respectively. The impact of pre-operative factors on PFS and OS, evaluated through the univariate model, is reported in Supplementary Table 1. After adjustment for covariates (Table 2), a worse OS was observed in patients aged ≥ 70 years (HR = 2.18; 95% CI 1.27–3.73), initial stage IV (HR = 2.37; 95% CI 1.18–4.76), DFI < 12 months (HR = 1.72; 95% CI 1.01–2.94), and loco-regional recurrence (HR = 2.22; 95% CI 1.22–4.04). These pre-operative factors were also associated with worse PFS, but DFI < 12 months was not statistically significant in the multivariable model. Cancer site was not significantly associated with OS, but patients with hypopharyngeal cancer reported a worse PFS than those with other cancer sites (HR = 1.89; 95% CI 1.01–3.53).

**Table 2:** Multivariable hazard ratio (HR)^a^ and corresponding 95% confidence intervals (CI) of PFS event and death for socio-demographic characteristics and pre-operative clinical features.

	N	Progression-free survival		Overall survival	
		HR (95% CI)	Wald χ^2^	HR (95% CI)	Wald χ^2^
**Gender**					
Male	122	Reference		Reference	
Female	42	0.83 (0.49–1.39)	P = 0.467	0.84 (0.48–1.47)	P = 0.531
**Age at salvage surgery (year)**					
< 60	61	Reference		Reference	
60–69	59	1.33 (0.81–2.19)	P = 0.253	1.21 (0.72–2.03)	P = 0.476
≥ 70	44	1.89 (1.13–3.15)	P = 0.015	2.18 (1.27–3.73)	P = 0.005
**Primary cancer site**					
Oral cavity	40	1.59 (0.86–2.93)	P = 0.140	1.36 (0.71–2.61)	P = 0.353
Oropharynx	54	0.79 (0.44–1.44)	P = 0.446	0.71 (0.38–1.34)	P = 0.294
Hypopharynx	25	1.89 (1.01–3.53)	P = 0.456	1.62 (0.83–3.16)	P = 0.159
Larynx	45	Reference		Reference	
**Initial T stage**					
T1–T2	76	Reference		Reference	
T3–T4	56	1.51 (0.84–2.70)	P = 0.166	1.63 (0.91–2.87)	P = 0.133
**Initial N stage**					
N0	60	Reference		Reference	
N1-N3	67	1.56 (0.78–3.12)	P = 0.211	0.98 (0.47–2.01)	P = 0.948
**Initial TNM stage**					
I-II	45	Reference		Reference	
III	34	1.64 (0.85–3.17)	P = 0.137	1.61 (0.79–3.28)	P = 0.193
IV	53	2.34 (1.23–4.46)	P = 0.010	2.37 (1.18–4.76)	P = 0.015
Unknown	32	1.59 (0.79–3.19)	P = 0.190	1.70 (0.82–3.53)	P = 0.158
**Initial treatment**					
Surgery ± RT/RCT	39	Reference		Reference	
RT ± CT	125	0.91 (0.49–1.67)	P = 0.750	0.90 (0.45–1.77)	P = 0.749
**Disease-free interval (months)**					
≥ 12	56	Reference		Reference	
< 12	91	1.36 (0.81–3.78)	P = 0.220	1.72 (1.01–2.94)	P = 0.048
**Recurrence site**					
Local	90	Reference		Reference	
Regional	48	1.36 (0.81–2.27)	P = 0.246	1.43 (0.82–2.49)	P = 0.213
Loco-regional	26	2.15 (1.22–3.78)	P = 0.008	2.22 (1.22–4.04)	P = 0.009

*RT* radiotherapy, *CT* chemotherapy, *CRT* chemo-radiotherapy.

^a^Estimated through Cox proportional hazard model, adjusting for gender, age, primary cancer site, initial TNM stage, disease-free interval and recurrence site.

### Post-operative prognostic factors

Post-operative factors were further investigated (Table 3). Negative margins were achieved in 106 (64.6%) patients; conversely, margins were microscopically positive in 16 (9.7%) patients, and close in 42 (25.6%) patients. Twenty-six patients (15.9%) experienced major complications. In particular, wound impairments with tissue necrosis or infection, pharyngocutaneus fistula and free flap failure were reported in 10 (38.5%), 8 (30.8%) and 3 (11.5%) patients, respectively. Major medical complications (i.e., stroke and pulmonary embolism) were reported in two patients. One patient died postoperatively due to massive hemorrhage. None of the post-operative factors was associated with PFS or OS in the multivariate model (Supplementary Tables 2 and 3).

**Table 3:** Hazard ratio (HR)^a^ and corresponding 95% confidence intervals (CI) of PFS event and death for surgical features.

	n	Progression-free survival		Overall survival	
		HR (95% CI)	Wald χ^2^	HR (95% CI)	Wald χ^2^
**Margins**^**b**^					
R0	105	Reference		Reference	
R1-R2	44	1.56 (0.87–2.79)	P = 0.139	0.99 (0.53–1.84)	P = 0.980
**Complications**					
No	138	Reference		Reference	
Yes	26	1.09 (0.64–1.87)	P = 0.746	1.14 (0.64–2.02)	P = 0.661
**Lymphovascular invasion**^**c**^					
No	65	Reference		Reference	
Yes	51	0.81 (0.45–1.46)	P = 0.484	1.04 (0.56–1.93)	P = 0.897
**Perineural invasion**^**b,c**^					
No	66	Reference		Reference	
Yes	19	1.17 (0.54–2.52)	P = 0.694	1.53 (0.69–3.37)	P = 0.295

^a^Estimated through Cox proportional hazard model, adjusting for gender, age, cancer site, initial TNM stage, disease free interval, and recurrence site.

^b^The sum does not add up to total because of missing values.

^c^Patients with regional or loco-regional recurrence.

### Prognostic scores

**Figure 1:**
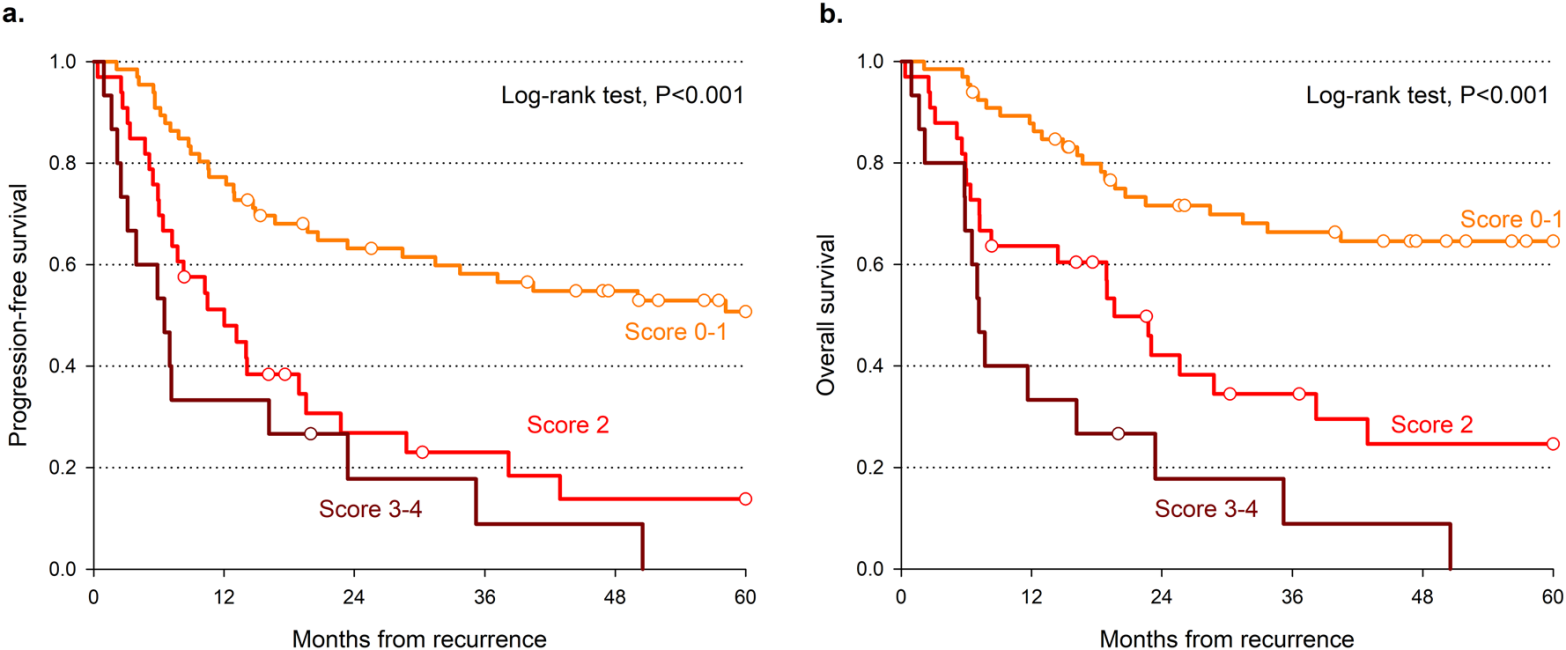
Progression free survival (**a**) and overall survival (**b**) according to prognostic score values. Survival curves were estimated according to Kaplan–Meier methods, and differences across score levels were evaluated through log-rank test.

**Figure 2:**
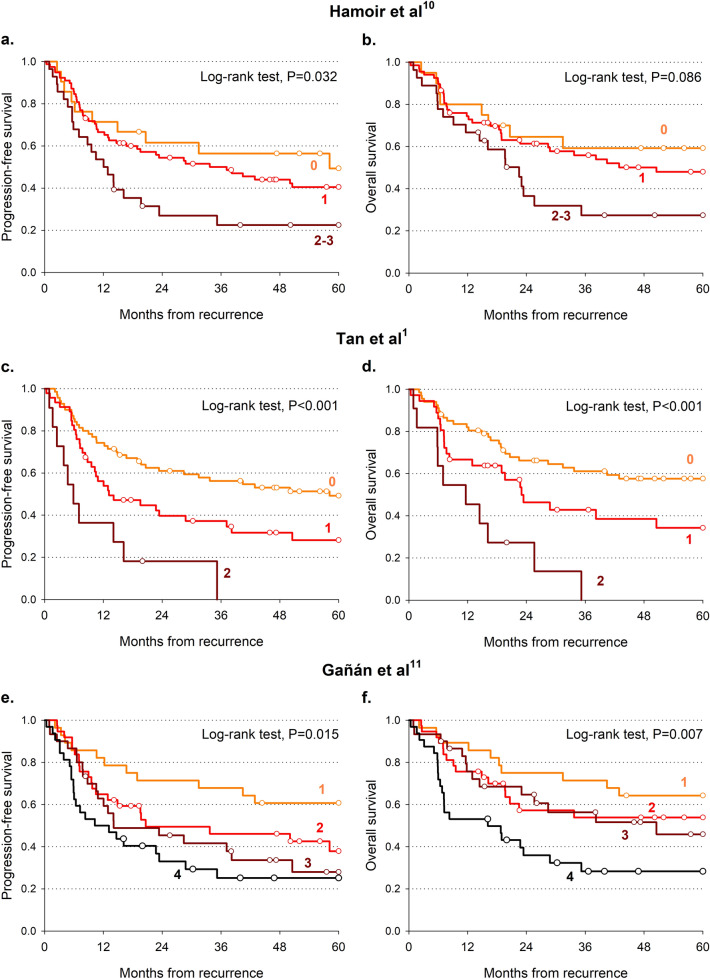
Progression-free survival (**a**,**c**,**e**) and overall survival (**b**,**d**,**f**) according to published prognostic scores. Survival curves were estimated according to Kaplan–Meier methods, and differences across score levels were evaluated through log-rank test.

A prognostic score was elaborated taking into account the four independent factors significantly associated with OS (namely, age > 70 years, initial stage IV, DFI < 12 months, and loco-regional recurrence). Patients with 0 and 1 scores and those with 3 and 4 scores reported similar OS, and they were therefore combined (Table 4). Patients with 0–1 score reported a 5-year OS of 65.7% compared to 35.3% for score = 2 and 0.0% for 3–4 score (P < 0.001; Fig. 1a, Table 4). A similar pattern emerged also for PFS (Fig. 1b, Table 4). The present score showed the greatest difference between the most favorable and the least favorable groups for both 5-year OS (C-index = 0.675) and PFS (C-index = 0.643). The score by Tan and colleagues^1^ reported similar results (Fig. 2c,d), with a C-index for OS of 0.616, whereas the scores by Hamoir and colleagues^12^ (Fig. 2a,b) and Gañán and colleagues^13^ (Fig. 2e,f) showed worse predictability.

**Table 4:** Comparison of progression-free survival and overall survival according to predictive scores.

Score (reference)	Predictors of poor prognosis	Score levels	Progression-free survival				Overall survival			
			2 years	5 years	HR (95% CI)^a^	Wald χ^2^	2 years	5 years	HR (95% CI)^a^	Wald χ^2^
Lupato et al	Age > 70 years Initial stage IV DFI < 12 months Loco-regional recurrence	0–1	64.3%	52.3%	Reference		72.5%	65.7%	Reference	
		2	36.2%	26.7%	2.18 (1.31–3.63)	P = 0.003	48.0%	35.3%	2.49 (1.40–4.42)	P = 0.002
		3–4	22.1%	0.0%	4.09 (2.19–7.63)	P < 0.001	22.1%	0.0%	5.61 (2.89–10.92)	P < 0.001
			C-index: 0.643 (0.585–0.702)				C-index: 0.675 (0.614–0.736)			
Hamoir et al.^12^	Initial laryngeal cancer Initial stage III-IV Loco-regional recurrence	0	61.5%	49.4%	Reference		66.3%	61.2%	Reference	
		1	54.4%	40.5%	1.28 (0.65–2.55)	P = 0.478	63.1%	51.8%	1.28 (0.59–2.75)	P = 0.535
		2–3	26.9%	22.5%	2.33 (1.09–4.97)	P = 0.029	35.2%	26.4%	2.42 (1.05–5.54)	P = 0.037
			C-index: 0.568 (0.508–0.629)				C-index: 0.562 (0.492–0.631)			
Tan et al.^1^	Initial stage IV Loco-regional recurrence	0	61.0%	49.2%	Reference		67.7%	59.5%	Reference	
		1	39.7%	28.2%	1.80 (1.10–2.94)	P = 0.019	49.9%	40.5%	1.74 (1.01–3.01)	P = 0.046
		2	0.0%	0.0%	4.39 (2.14–9.03)	P < 0.001	27.3%	0.0%	4.68 (2.23–9.80)	P < 0.001
			C-index: 0.609 (0.549–0.669)				C-index: 0.616 (0.547–0.680)			
Gañán et al.^13^	Initial laryngeal cancer Initial N + Inital surgical treatment DFI < 12 months	1	71.4%	60.7%	Reference		75.0%	64.3%	Reference	
		2	49.5%	37.9%	1.84 (0.88–3.82)	P = 0.103	57.2%	53.9%	1.48 (0.67–3.27)	P = 0.332
		3	45.4%	28.0%	2.33 (1.11–4.88)	P = 0.025	64.7%	45.9%	1.61 (0.71–3.63)	P = 0.253
		4	33.0%	25.2%	3.09 (1.50–6.38)	P = 0.002	36.0%	28.3%	3.19 (1.50–6.78)	P = 0.003
			C-index: 0.608 (0.542–0.674)				C-index: 0.615 (0.545–0.685)			

*DFI* disease-free interval.

^a^Estimated through Cox proportional hazard model.

The score predictability was further evaluated according to cancer site (Table 5). Patients with three or four unfavorable factors showed a significantly higher risk of death for cancer of the oral cavity (HR = 3.77; 95% CI 1.03–13.76) and oropharynx (HR = 9.26; 95% CI 1.88–45.65), whereas the excess risk was not significant for hypopharyngeal cancer (HR = 2.92; 95% CI 0.82–10.41). No patients with laryngeal cancer reported 3–4 score; those with two unfavorable factors reported a fivefold higher death risk (HR = 5.07; 95% CI 1.76–14.55) than those with zero or one unfavorable factor. Similar pattern emerged for PFS. However, the low number of patients in each subgroup calls for caution in drawing conclusions.

**Table 5:** Predictive score according separate cancer site.

Cancer site	Score levels	Patients	Progression-free survival		Overall survival	
			HR (95% CI)^a^	Wald χ^2^	HR (95% CI)^a^	Wald χ^2^
Oral cavity	0–1	24	Reference		Reference	
	2	7	1.18 (0.39–3.58)	P = 0.765	1.96 (0.61–6.27)	P = 0.259
	3–4	3	2.59 (0.74–9.07)	P = 0.136	3.77 (1.03–13.76)	P = 0.045
Oropharynx	0–1	10	Reference		Reference	
	2	23	3.05 (0.87–10.69)	P = 0.081	3.09 (0.68–14.00)	P = 0.143
	3–4	8	6.04 (1.54–23.76)	P = 0.010	9.26 (1.88–45.65)	P = 0.006
Hypopharynx	0–1	8	Reference		Reference	
	2	7	0.95 (0.32–2.85)	P = 0.927	1.15 (0.33–4.03)	P = 0.827
	3–4	6	2.15 (0.67–6.97)	P = 0.201	2.92 (0.82–10.41)	P = 0.099
Larynx	0–1	30	Reference		Reference	
	2	6	4.85 (1.72–13.65)	P = 0.003	5.07 (1.76–14.55)	P = 0.003
	3–4	0	–		–	

^a^Estimated through Cox proportional hazard model.

## Discussion

Although salvage surgery represents the best choice in resectable recurrent HNSCC, it often results in poor outcome in terms of survival and quality of life^3^. Combining four pre-operative factors independently associated with OS (namely, age ≥ 70 years, stage IV, DFI < 12 months, and loco-regional recurrence), the results of this study provide helpful indications to identify patients who would benefit the most from salvage surgery. Alternative treatments could be considered for patients with at least three unfavorable predictors.

In the last years, other interesting prognostic algorithms have been evaluated. Tan and colleagues^1^ created a score using initial stage IV and concurrent loco-regional failure. Similar to our score, they have successfully identified patients with the worst prognosis (the 2-year OS for the presence of both conditions was 0%), but the difference in the low and medium risk groups was less marked. Subsequently, by using a large series of 1088 patients treated with salvage surgery for relapsed HNSCC, Gañán and colleagues^13^ conducted a recursive partitioning analysis that defined four groups of patients considering as prognostic factors the initial stage, the tumor site, the initial treatment, and the DFI. They found that the possibility of carrying out potentially curative treatment varied between 15 and 81% depending on those factors. More recently, Hamoir and colleagues^12^ have proposed a prognostic score that included three pre-operative factors: loco-regional recurrence, initial laryngeal cancer, and initial stage III–IV.

It is worth noting that all the considered scores^1,12,13^ included loco-regional recurrence and advanced initial stage as predictors, demonstrating the strong prognostic significance of these factors. Consistently, in our study, patients with loco-regional recurrence and initial stage IV reported the highest risk of recurrence and death. The presence of nodal metastases is commonly accepted as the single worst prognostic factor at first presentation that halves survival^15^. Similarly, recurrence both on the primary site and on regional nodes associated with a poor outcome in several studies on different HNSCC cancer sub-sites^9,16–19^. In our study, 50% of HNSCC patients with loco-regional recurrence further relapsed within 2 years after salvage surgery, and 40% of them ultimately died. Although the prognostic value of both initial and recurrent tumor stage is still under debate, several studies have reported a high incidence of loco-regional recurrence and distant metastasis after salvage surgery in patients with advanced initial stage tumors^1,20–23^. Interestingly, Goodwin and colleagues^9^ have described a relevant difference in two-year disease-free survival based on recurrent stage. Otherwise, this relationship has not been observed in other studies^12,20^.

In line with Gañán and colleagues^13^, our data indicated that a short DFI might represent an independent prognostic factor. Despite other studies associated a short DFI with a poor outcome^24–28^, there is no consensus on the time interval to be considered as cutoff. Indeed, Kim and colleagues^24^ considered a cutoff of 6 months, Liao and colleagues^25^ a cutoff of 10 months, in our series the cutoff was 12 months. Interestingly, late recurrence has been reported as a favorable prognostic factor in cancers of the oral cavity^27^, oropharynx^28^, and larynx^29^.

Besides DFI, we also included age that allowed the identification of the group of patients with the best 5-year OS. In particular, age > 70 years appeared as predictive factor for a poor prognosis. Interestingly, Kim and colleagues^24^ have highlighted the importance of age and comorbidities as prognostic factors in patients treated with salvage surgery. In fact, they observed that medical comorbidities and age measured by Charlson-Age Comorbidity Index, primary T3 or T4 stage, and short DFI represented independent risk factors for death within one year after salvage surgery. Unfortunately, data about comorbidities were not available in our study.

For the sake of comparison, we analyzed our data using the scores of the cited studies^1,12,13^. Our score reported the highest predictability on OS, similar to the score by Tan and colleagues (C-index = 0.616). Both scores used initial stage and loco-regional recurrence as predictors, but the inclusion of age and DFI in our score allowed the identification of the group of patients with the best 5-year OS. The scores by Hamoir and colleagues^12^ and Gañán and colleagues^13^ showed worse predictability. Further, our score and the one by Tan and colleagues^1^ are the two that better identify groups with different risk of death and PFS event.

The score was further evaluated in specific cancer sites. Although the low number of cases calls caution in interpreting the results, the score showed good capability to identify patients at high risk of death for all cancer sites. This was not totally unexpected. Indeed, despite the differences in clinical presentation across original cancer site, a recent meta-analysis of clinical outcomes after salvage surgery for recurrent advanced stage HNSCC^30^ found no difference according to sub-sites. Nonetheless, further investigations should be conducted to fill the gap in site-specific predictors.

Considering our score, an alternative treatment to salvage surgery could be considered for patients with two or more unfavorable pre-operative factors. Radiation therapy, with or without concurrent chemotherapy, is a now feasible option due to the improvement of radiation technique^31^. Initial trials on conformal radiotherapy (RTOG 9610 and RTOG 9911) reported high rate of acute and late toxicity and treatment related deaths, with a 2-year OS below 25%^32,33^. The advent of intensity modulated radiotherapy (IMRT) and stereotactic body radiotherapy (SBRT) allows the reduction of dose to organs at risk (OARs), thus reducing toxicity, maintaining while improving the target coverage^34^. Recently, few studies on patients re-irradiated with protons and carbon ions showed encouraging results in terms of OS, loco-regional control, and low incidence of toxicities, but they mostly concern re-irradiation of base skull and nasopharyngeal cancer^35,36^. Nonetheless, bleeding represented the most important cause of radiation-related death^35,36^, which suggests surgery as the preferable salvage treatment.

Systemic therapy, such as chemotherapy and immunotherapy, can also be considered as a salvage treatment in recurrent HNSCC patient. EXTREME regimen was the gold standard in the pre-immunotherapy era^37^. Nowadays, immunotherapy, with or without concomitant chemotherapy, seems a promising approach in this setting^38,39^, with an improvement of OS compared to standard EXTREME regimen^39^. The benefit of immunotherapy is nonetheless limited, with response rate below 20%^38,39^. Therefore, a careful evaluation of patients candidate for immunotherapy should be conducted using appropriate biomarkers (e.g., PD-L1 expression), also taking into consideration the resource demand of such treatments. Consequently, surgery should still be considered as the elective treatment in locally recurrent HNSCC patients, but further trials should investigate the optimal combination of modern treatment approaches. Lastly, comprehensive palliative and supportive care should be considered for recurrent patients who are not candidate for any salvage treatment.

Some limitations have to be acknowledged. First, the present score was created using a single-center retrospective cohort, so that external validation is lacking. In particular, the comparison of different scores on the same cohort used for score creation has to be considered with caution. Moreover, patients included in the present cohort were heterogeneous in terms of initial cancer site and treatment. The inclusion of recurrent cancers for different primary sub-sites may have raised the heterogeneity of results by increasing the complexity of their interpretation; however, at the same time, it enlarged the sample size, allowing the estimation of the independent prognostic value of several pre-operative factors. It is worth noting that the factors identified by the preset study were consistent with those reported by studies on oral cancer^26,27^ and oropharyngeal cancer^28^. Further, the present study considered patients with local, regional, and loco-regional recurrent HNSCCs, which may imply heterogeneous surgical treatments with different complexity. This choice has increased the heterogeneity of our cohort but, at the same time, it has allowed the evaluation of site of recurrence as a predictor. The large sample size in comparison to previous studies on salvage surgery for recurrent HNSCCS should be accounted among the strengths of the present study. Further, all patients were treated by the same multidisciplinary group that used internal recommendations based on the international guide-lines, and with homogeneous treatment modality techniques.

In conclusion, four easily identifiable pre-operative factors (age > 70 years, initial stage IV, DFI > 12 months, loco-regional failure) were independently associated with OS. The combination of these factors into a single prognostic score may be useful for clinicians to improve the selection of patients candidates to salvage surgery. Patients with two or more of these factors should be clearly informed about the low success rate after salvage surgery. Alternative treatments, such as re-irradiation, chemotherapy or immunotherapy, should be considered.

## Supplementary information

Supplementary Information

## Data Availability

Data are available for research purpose upon request to the corresponding author.
