# Supplementary material for: A pre-operative prognostic score for the selection of patients for salvage surgery after recurrent head and neck squamous cell carcinomas
Source: Sci Rep. 2021 Jan 12;11:502. doi: 10.1038/s41598-020-79759-0 (PMC7804332; doi:10.1038/s41598-020-79759-0)
Supplement: Supplementary file 1 — Supplementary Information [file 41598_2020_79759_MOESM1_ESM.pdf]

Supplementary Information

A pre-operative prognostic score for the selection of patients for salvage surgery after recurrent head and neck squamous cell carcinomas

Valentina Lupato[1], Jerry Polesel[2], Fabio Biagio La Torre[1], Giuseppe Fanetti[3*], Elisabetta Fratta[4], Carlo Gobitti[3], Gustavo Baldassarre[5], Emanuela Vaccher[6], Giovanni Franchin[3], Vittorio Giacomarra[1]

Supplementary Table 1. Univariate hazard ratio (HR)[a] and corresponding 95% confidence intervals (CI) of PFS event and death for socio-demographic characteristics and pre-operative clinical features.

Supplementary Table 2. Univariate hazard ratio (HR)[a] and corresponding 95% confidence intervals (CI) of PFS event and death for surgical features.

Supplementary Table 1. Univariate hazard ratio (HR)[a] and corresponding 95% confidence intervals (CI) of PFS event and death for socio-demographic characteristics and pre-operative clinical features.

	n	Progression-free survival		Overall survival	
		**HR (95% CI)**	**Wald χ^2**	**HR (95% CI)**	**Wald χ^2**
Gender					
Male	122	Reference		Reference	
Female	42	0.79 (0.51-1.24)	P=0.304	0.74 (0.46-1.20)	P=0.224
Age at salvage surgery (year)[a]					
<60	61	Reference		Reference	
60-69	59	1.14 (0.73-1.79)	P=0.572	1.01 (0.62-1.63)	P=0.977
≥70	44	1.70 (1.06-2.75)	P=0.030	1.92 (1.17-3.17)	P=0.010
Primary cancer site					
Oral cavity	40	1.84 (1.00-3.40)	P=0.051	1.47 (0.78-2.78)	P=0.238
Oropharynx	54	1.44 (0.85-2.43)	P=0.176	1.38 (0.81-2.37)	P=0.239
Hypopharynx	25	2.92 (1.65-5.18)	P<0.001	2.56 (1.39-4.71)	P=0.002
Larynx	45	Reference		Reference	
Initial tumor size					
T1-T2	76	Reference		Reference	
T3-T4	56	2.24 (1.42-3.54)	P<0.001	2.65 (1.92-4.31)	P<0.001
Initial lymph nodes status					
N0	60	Reference		Reference	
N1-N3	67	2.05 (1.31-3.21)	P=0.002	1.74 (1.08-2.80)	P=0.024
Initial TNM staging					
I-II	45	Reference		Reference	
III	34	2.24 (1.22-4.10)	P=0.009	2.16 (1.12-4.17)	P=0.021
IV	53	2.95 (1.71-5.09)	P<0.001	3.06 (1.70-5.50)	P<0.001
Unknown	32	1.96 (1.08-3.55)	P=0.027	2.37 (1.27-4.42)	P=0.007
Initial treatment					
Surgery ± RCT	39	Reference		Reference	
RT ± CT	125	0.95 (0.60-1.53)	P=0.844	1.09 (0.65-1.81)	P=0.752
Disease-free interval (months)					
≥12	56	Reference		Reference	
<12	91	1.64 (1.05-2.56)	P=0.030	2.11 (1.29-3.46)	P=0.003
Recurrence site					
Local	90	Reference		Reference	
Regional	48	1.63 (1.05-2.53)	P=0.031	1.75 (1.10-2.80)	P=0.019
Loco-regional	26	2.45 (1.47-4.09)	P<0.001	2.57 (1.51-4.38)	P<0.001

[a]Estimated through Cox proportional hazard model, adjusting for gender and age.

Supplementary Table 2. Univariate hazard ratio (HR)[a] and corresponding 95% confidence intervals (CI) of PFS event and death for surgical features.

	n	Progression-free survival		Overall survival	
		**HR (95% CI)**	**Wald χ^2**	**HR (95% CI)**	**Wald χ^2**
Margins[b]					
R0	105	Reference		Reference	
R1-R2	44	2.00 (1.29-3.09)	P=0.002	1.64 (1.02-2.62)	P=0.041
Complications					
No	138	Reference		Reference	
Yes	26	1.46 (0.87-2.40)	P=0.151	1.54 (0.90-2.63)	P=0.114
Lymphovascular invasion[c]					
No	65	Reference		Reference	
Yes	51	1.48 (0.91-2.43)	P=0.110	1.65 (0.97-2.81)	P=0.063
Perineural invasion[b,c]					
No	66	Reference		Reference	
Yes	19	1.28 (0.67-2.44)	P=0.463	1.63 (0.83-2.00)	P=0.153

[a]Estimated through Cox proportional hazard model, adjusting for gender and age. [b]The sum does not add up to total because of missing values. [c]Patients with regional or loco-regional recurrence.
